# H-mode transition and pedestal studies

**DOI:** 10.1098/rsta.2021.0241

**Published:** 2023-02-20

**Authors:** Yasmin Andrew, Eun-jin Kim

**Affiliations:** ^1^ Blackett Laboratory, Imperial College London, London SW7 2BW, UK; ^2^ Fluid and Complex System Research Centre, Coventry University, Coventry CV1 2TT, UK

**Keywords:** pedestal, H-mode, L-H transition

## Abstract

The high confinement mode (H-mode) is the widely adopted standard operation scenario for the path to fusion in toroidal confinement devices. Since its discovery in 1982, the H-mode and access to the H-mode (the low to high and high to low transitions) remain two of the most actively researched areas in magnetically confined fusion programmes across the world. Significant progress has been made in the understanding of the intricate H-mode phase dynamics in recent years, from improvement in experimental diagnostic capability, theoretical development and modelling. The ‘H-mode transition and pedestal studies’ Special Issue provides a timely overview of recent progress in the study of H-modes covering experimental studies, further theoretical inquiry and computational modelling.

This article is part of a discussion meeting issue ‘H-mode transition and pedestal studies in fusion plasmas’.

## 1. Overview

Understanding and controlling plasma transport lies at the very heart of successful magnetically confined fusion. Many precursors to the success of next step fusion device operation remain, and important plasma physics questions are unanswered; plasma transport and confinement continue to be actively studied in different fusion devices internationally, including the tokamak, the stellarator, other spherical tori and field-reversed configuration machines. The plasma transition from the low to high (L-H) confinement regime, one of the most remarkable discoveries in fusion history, refers to the sudden improvement of confinement when input power is increased above a critical value [1]. The L-H transition is accompanied by the formation of a pedestal in the confined plasma edges, a relatively narrow region with significantly enhanced pressure profile gradients and reduced transport. This phenomenon has been reliably reproduced in different fusion devices since its first discovery in the 1980s [2].

The studies of the L-H transition, the backwards H-L transition and the H-mode pedestal have remained some of the most important research topics ever since. Questions on how to enter and exit the H-mode on ITER in the preliminary hydrogen and helium plasmas, and the later planned deuterium plasmas, with the available heating power, remain a crucial part of practical machine operation planning [3]. This H-mode topic is a rapidly evolving field, with a growing number of planned conventional and spherical cross-machine studies with upgraded suites of edge, core and divertor plasma diagnostics and advanced analysis techniques.

This themed issue aims to present valuable and pertinent research outcomes, contributing to ongoing worldwide H-mode access and H-mode studies. It comprises articles on new analysis of experimental studies, exciting theoretical developments and comprehensive reviews of the topics, providing an outlook for current strands of global research into the H-mode and H-mode access. The following provides a brief description of each article in the order that they appear in this Special Issue.

Thyagaraja *et al.* [4] report CUTIE simulation results on edge localized mode (ELM) control by resonant magnetic perturbations (RMP) fields, highlighting the bifurcation phenomenon in the RMP amplitude in ELM suppression; Andrew *et al.* [5] present the first experimental results of transition from L-mode to dithering H-modes on the compact, spherical tokamak ST40 and extend the L-H transition predator–prey model to include the contribution from MHD instabilities and pedestal pressure gradient. A comprehensive review of the role of sheared plasma flow on the evolution of confinement from the L-H transition to the H-mode density limit is provided by Diamond *et al.* in [6]. Yan *et al.* [7] provide detailed observations of edge plasma turbulence flow and analyse their effect on the transition to H-mode on the DIII-D experiment. The correlation between turbulence flow and E×B velocities across the L-H transition on DIII-D is analysed in depth in [8] for comparable deuterium and hydrogen shots. The review by Kishimoto *et al.* [9] summarizes comprehensive investigation of large-scale transport dynamics and avalanches in L-mode turbulence obtained from flux-driven gyrokinetic simulations. Kim & Hollerbach [10] present a time-dependent, non-perturbative statistical method of describing ELMs, highlighting the effect of stochastic noise on phase-mixing and mitigation of extreme bursts of large ELMs. A rigorous comparison between measured experimental pedestal structure in JET-ILW plasmas and an electron temperature gradient model for turbulent heat transport is presented by Field *et al.* [11]. Teaca *et al.* [12] investigate the self-organization nature of a sheared plasma flow as transitions between (turbulent and laminar) states in a reduced fluid (interchange) model. The theme issue closes with a comprehensive analysis of H-mode transitions on the TJII Stellarator by Estrada *et al.* [13]

## Data Availability

This article has no additional data.
